# Good Biocompatibility and Sintering Properties of Zirconia Nanoparticles Synthesized via Vapor-phase Hydrolysis

**DOI:** 10.1038/srep35020

**Published:** 2016-10-11

**Authors:** Jigang Wang, Wenyan Yin, Xiao He, Qiang Wang, Ming Guo, Shaowei Chen

**Affiliations:** 1 Laboratory for Micro-sized Functional Materials & College of Elementary Education, Capital Normal University, Beijing, 100048, PR China; 2 Department of Chemistry, Capital Normal University, Beijing, 100048, PR China; 3 Key Laboratory for Biomedical Effects of Nanomaterials and Nanosafety Institute of High Energy Physics, Chinese Academy of Sciences, Beijing, 100049, China; 4 Department of Chemistry and Biochemistry, University of California, Santa Cruz, CA 95064, USA

## Abstract

ZrO_2_ nanoparticles were synthesized by a vapor-phase hydrolysis process, and characterized in terms of crystalline structures, hardness and microstructures by X-ray diffraction, Vickers hardness test method, and atomic force microscopy (AFM) measurements. Moreover, *in vitro* cytotoxicity evaluation and hemolysis assay showed that the nanoparticles possessed good biocompatibility. Hardness investigations and AFM measurements indicated that both the sintering temperature and compression force played an important role in determining the physical behaviors (hardness, roughness and density) of flakes of the ZrO_2_ nanoparticles. When ZrO_2_ nanoparticles synthesized at 500 °C were pressed into flakes under 6 MPa and sintered at 1400 °C, the resulting flakes exhibited an optimal combination of hardness (534.58 gf·mm^−2^), roughness (0.07 μm) and density (4.41 g·cm^−3^). As the Vickers hardness value of human bones is of 315~535 gf·mm^−2^ and the density of adult femuris about 1.3~1.7 g·cm^−3^, the experimental results showed that the ZrO_2_ flakes were comparable to human bones with a higher density. As a result, the synthesized ZrO_2_ NPs may be useful for biomedical applications, especially for bone repair and replacement in future.

The field of biomaterial technology has rapidly progressed over the last few decades with the advent of advanced medical devices and implants developed from metals and ceramic^1^. It is known that implantation of biomaterials also causes a cascade of reactions in the biological environment. For instance, the insertion of some implants may lead to bacterial infections along the bone/material interface due to poor biocompatibility of the implants despite total disinfection prior to the surgeries. After the bacterial proliferation period, the biomaterial-associated infections can hardly be cured by traditional systemic antibiotic therapy^1^,^2^. Hence, there is a pressing need for the development of safe biocompatible implants. The success of rapid osseointegration of orthopedic implanted materials is dependent on the formation capability between the implants and bones, when the implants are embedded in aliving body^3^. Metal alloys (e.g., Ti, Sr, Co, Cr, etc.) are generally expected to be bioinert with biological systems in the human body. Consequently, implants made of metal alloys used in bone reconstruction typically lack desired osseointegration properties, which limits their biological fixation with bone tissues and consequently long-term *in vivo* stability^4^. Moreover, metal ions can be released from alloy implants due to its frequent interactions with the surrounding physiological environment, which may lead to detrimental effects on the long-term health of the patients^1^,^2^,^3^.

Zirconia (ZrO_2_), as one of the most important oxide materials, exhibits excellent properties including low thermal conductivity, high thermal expansion, good thermal stability, fine mechanical strength, good fracture toughness and high thermal shock resistance^5^,^6^,^7^. Thus, ZrO_2_ has been used in abroad range of applications as catalysts/catalyst support^8^,^9^,^10^,^11^,^12^,^13^,^14^, oxygensensors^11^,^12^, fuelcells^13^,^14^, biological materials^15^,^16^, automobile parts and thermal barrier coatings on metal components^17^,^18^,^19^. ZrO_2_ nanomaterials have also been employed in medical and orthopedic applications, mainly for repair and replacement of diseased and damaged parts of human skeleton, bones, teeth and joints due to their good biocompatibility, osseointegration, and bioinertness^20^. In fact, because of nontoxicity to the surrounding tissues, implants based on ZrO_2_ nanoparticles (NPs) have been utilized for clinical total hip replacements, and as a prevalent biomaterial in prosthetic dentistry and dental implantology^5^,^13^,^16^,^21^,^22^. This is largely ascribed to the good physical performance of sintered ZrO_2_ devices, in terms of hardness, density, roughness and stability.

Currently, the synthetic methods of nano-sized ZrO_2_ mainly include gas-phase methods (e.g., gas-phase chemical synthesis^23^,^24^,^25^,^26^ and chemical vapor deposition^27^,^28^,^29^), and liquid-phase methods (fast precipitation^30^,^31^,^32^,^33^, sol–gel^34^,^35^,^36^, solvent evaporation, and hydrothermal treatment). However, up to now, reports remain scarce involving a comprehensive and detailed investigation of the sintering properties of ZrO_2_ NPs under different conditions.

In this paper, ZrO_2_ NPs were synthesized by using a simple vapor-phase hydrolysis process at controlled temperatures, and utilized to prepare nanoflakes by compression and sintering. By a systematic variation of the compression force and sintering temperature, the hardness and density of the resulting ZrO_2_ nanoflakes were maximized whereas the roughness was minimized. The physical properties such as hardness, roughness as well as density of the sintered ZrO_2_ nanoflakes were better than those of the human bone. Furthermore, *in vitro* cytotoxicity and hemolysis evaluation showed that the ZrO_2_ NPs possessed good biocompatibility. These findings suggest great potential of the ZrO_2_ NPs as a biomcompatible material for medical implants for bone tissue engineering because they meet the demand of high physical properties of artificial hard tissues.

## Results

### Structures of ZrO_2_ NPs

**Figure 1:**
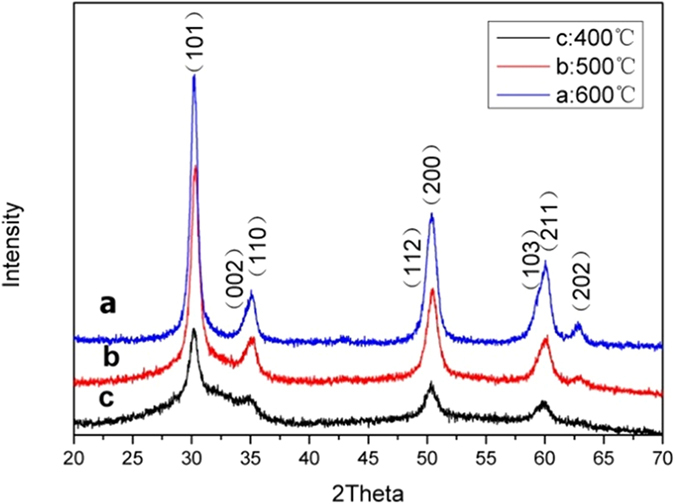
XRD patterns of ZrO_2_ NPs synthesized at different temperatures.

The structures of the obtained ZrO_2_ NPs were first characterized by X-ray diffraction (XRD) measurements. Figure 1 shows the XRD patterns of ZrO_2_ NPs synthesized at different temperatures (400, 500 and 600 °C). A series of well-defined peaks can be identified at 2θ = 30.2, 35.0, 50.4, 60.0 and 62.7°, which were ascribed to the diffractions of the (101), (110), (200), (211), and (202) crystalline planes of cubic phase ZrO_2_ (JCPDS card no. 49-1642)^21^, respectively. In addition, the asymmetric line shape of the peaks at 35.0, 50.4 and 60.0° suggested the formation of a tetragonal phase. The shoulder at 2θ = 34.5° was the diffraction of the (002) crystalline plane of tetragonal phase ZrO_2_ (JCPDS card no. 42-1164), and those at 2θ = 50.2° and 59.1° corresponding to the diffractions of the (112) and (103) crystalline planes^19^. Furthermore, from Fig. 1, it can be seen that the crystallinity of the ZrO_2_ NPs increased with increasing synthesis temperature from 400 °C to 600 °C.

### SEM Analysis

Further structural insights were obtained in SEM measurements. From Supporting Figure S1, it can be seen that the ZrO_2_ NPs are mostly in the range of 15 to 65 nm in diameter. Statistical analysis based on more than 50 particles showed that the average diameter of the nanoparticles decreased with increasing synthesis temperature, 40 nm at 400 °C, 35 nm at 500 °C, and 30 nm at 600 °C, as manifested in the core size histograms (Supporting Figure S2).

### *In Vitro* Cytotoxicity

**Figure 2:**
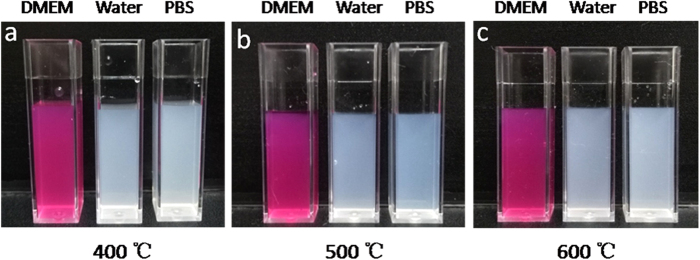
Photographs of ZrO_2_ NPs dispersions in DMEM, Water, PBS. ZrO_2_ NPs were synthesized at different temperatures (**a**) 400 °C, (**b**) 500 °C, and (**c**) 600 °C.

**Figure 3:**
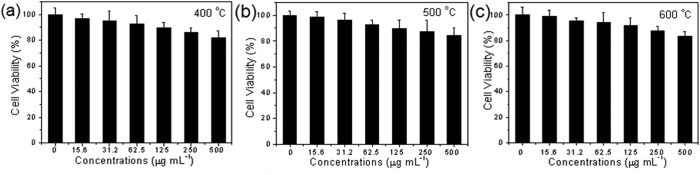
Cell viabilities of HUVEC cells after treatment with ZrO_2_ NPs at various concentrations for 24 h.

Interestingly, the resulting ZrO_2_ NPs were found to exhibit low cytotoxicity, as manifested in *in vitro* studies with human umbilical vein endothelial cells lines (HUVEC). Experimentally, ZrO_2_ NPs were dispersed under sonication at varied concentration (up to 1 mg·mL^−1^) into dulbecco’s modified eagle medium (DMEM) and added to the HUVEC cell culture. The *in vitro* cytotoxicity of the ZrO_2_ NPs in HUVEC cells was evaluated by CCK-8 assay. Control experiments were also carried out by dispersing the ZrO_2_ NPs in deionized water, and phosphate buffer saline (PBS) (Fig. 2). From Fig. 3, it can be seen that the ZrO_2_ NPs (up to 500 μg·mL^−1^) exerted virtually no effect on cell viability after co-incubation for 24 h. For example, the HUVEC cells retained 92% of viability even at the concentration of 500 μg·mL^−1^ of ZrO_2_ NPs synthesized at 400, 500, or 600 °C.

**Figure 4:**
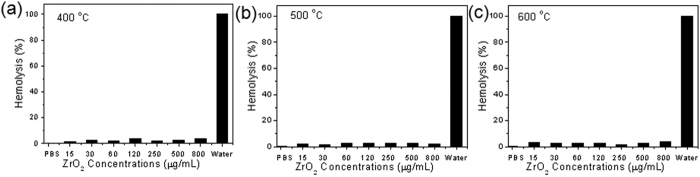
Hemolytic percent of RBCs. The RBCs was incubated with ZrO_2_ NPs at various concentrations for 4 h, using deionized water and PBS as positive and negative controls, respectively.

Hemolysis of the ZrO_2_ NPs was also evaluated by incubating the NPs with red blood cells (RBCs) for 4 h. It can be seen that the hemolytic percentages of RBCs were lower than 3.6% for the NPs synthesized at 400, 500, and 600 °C even at the concentration as high as 800 μg·mL^−1^, implying that these NPs had a negligible hemolytic activity (Fig. 4a–c). Therefore, it can be concluded that the ZrO_2_ NPs exhibit good biocompatibility and thus can act as a promising bio-ceramic materials for prosthetic dentistry and dental implantology^37^,^38^.

### Physical Hardness

With such remarkable cytocompatibility and hemocompatibility, ZrO_2_ NPs-based materials may be viable candidates for biomedical applications. Thus, the ZrO_2_ NPs were pressed into nanoflakes and subjected to sintering at elevated temperatures. Significantly, the obtained nanoflakes exhibited remarkable Physical characteristics. First, Vickers hardness tests were performed to examine the materials hardness, which was quantified by the peak load (P_max_) and projected contact area (A), 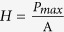.

The results were listed in Table 1. It can be seen that for ZrO_2_ NPs prepared at 400 °C, at the same sintering temperature, there is a maximum hardness that varies with the compression force (Supporting Figure S3); and at the same compression force, there is also a maximum hardness that varies with the sintering temperature. The maximum hardness (603.25 gf·mm^−2^) could be found at the compression force of 3 MPa and sintering temperature of 1200 °C. Similarly, for the ZrO_2_ NPs prepared at 500 and 600 °C, the maximum hardness can be identified at 3 MPa and 1200 °C, and 3 MPa and 1400 °C, respectively.

As is known, when the ZrO_2_ NPs are pressed into flakes, at low compression forces the flakes are likely crack-free such that the hardness increases with increasing compression force; however, at too high a compression force, (micro) cracks would start to form in the flakes, leading to reduced hardness. Likewise, at relatively low sintering temperatures, thermal stress in the particle cores was minimal and the flake hardness increased with increasing sintering temperature; in contrast, thermal stress became increasingly significant during high-temperature calcinations which led to the formation of cracks and hence reduced hardness^39^,^40^. Taken together, these results suggest that both the compression force and sintering temperature play an important role in the determination of the hardness of the flakes, and the maximum hardness may be manipulated by sintering temperature and compression force.

### Surface Roughness

**Figure 5:**
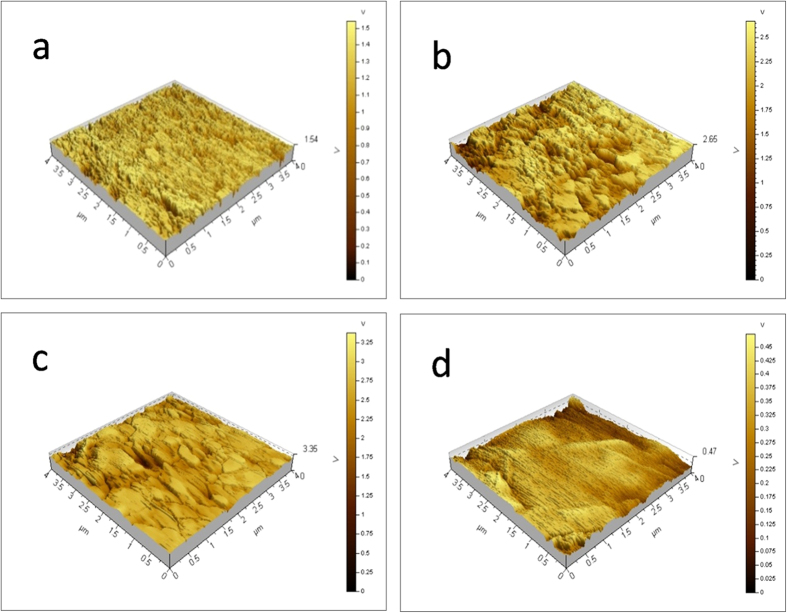
AFM images of ZrO_2_ flakes. The ZrO_2_ flakes were prepared by compression at 18 MPa of ZrO_2_ NPs synthesized at 400 °C and sintering at various temperatures: (**a**) 800 °C, (**b**) 1000 °C, (**c**) 1200 °C and (**d**) 1400 °C.

The surface morphologies of the ZrO_2_ nanoflakes were then analyzed by AFM measurements. From the AFM topographs in Fig. 5, the root mean square (rms) roughness of the ZrO_2_ nanoflakes was evaluated as a function of sintering temperature and compression force^41^. Experimentally, the rms roughness was quantified by taking an average of five data points measured over an area of 4 μm × 4 μm (Table 2). It can be seen that under the same compression force, the surface roughness of the flakes diminished with increasing sintering temperature (Supporting Figure S4).

**Figure 6:**
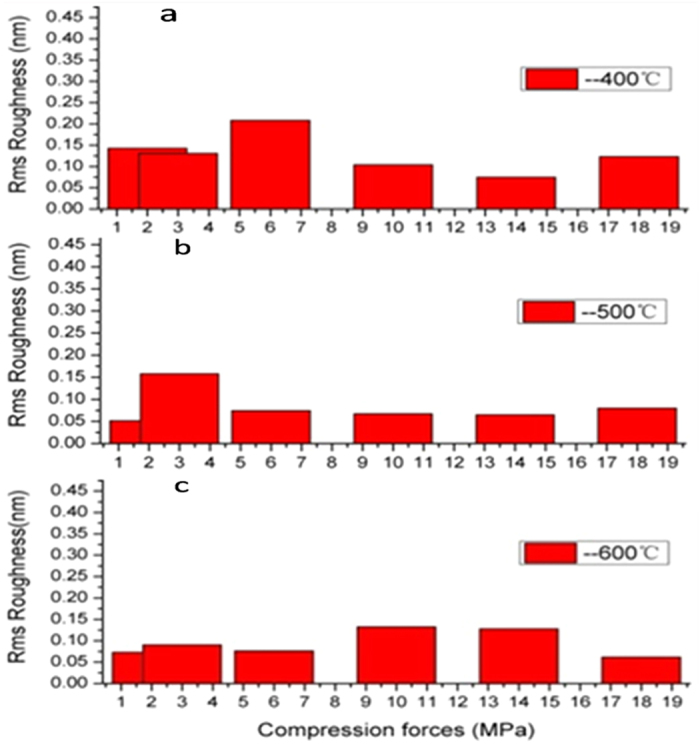
Surface roughness of ZrO_2_ flakes. The ZrO_2_ flakes prepared by sintering at 1400 °C under different compression forces of ZrO_2_ NPs synthesized at different temperatures (**a**) 400 °C, (**b**) 500 °C, and (**c**) 600 °C.

From Table 2, it can also be seen that at the same sintering temperature, the roughness of the flakes remained almost invariant with the compression force. Yet, at a constant compression force, the roughness of the flakes decreased with increasing sintering temperature, consistent with the results in Fig. 5. Interestingly, at the same sintering temperature, the ZrO_2_ surface roughness remained almost constant (Fig. 6), independent of the compression force. With increasing sintering temperature, the ZrO_2_ NPs tended to agglomerate and the surface roughness decreased accordingly. It has been reported that the surface properties play a critical role in the stability and function of bone-rebuilding materials^39^. Thus, changing the sintering temperature to reduce flakes roughness may enhance the biomedical applications of the ZrO_2_ flakes.

### Flake Density

Density is another important parameter in the assessment of materials for biomedical implants. The density of the ZrO_2_ nanoflakes was quantitatively evaluated by using a precision electronic hydrometer^42^,^43^, and the results are summarized in Table 3.

One can see that the density of the flakes varied with the sintering temperature and compression force, ranging from 2 to 7 g·cm^−3^, much higher than that of adult femurs (1.3~1.7 g·cm^−3^)^44^,^45^.

From the above studies, one can see that when ZrO_2_ NPs synthesized at 500 °C were pressed into flakes under the compression force of 6 MPa and sintered at 1400 °C, the resulting flakes exhibited an optimal combination of hardness (534.58 gf·mm^−2^), roughness (0.07 μm) and density (4.41 g·cm^−3^), considering that the highest hardness value for human bones is between 4 to 5 gf·mm^−2^, corresponding to Vickers hardness between 315 to 535 gf·mm^−2^, whereas the density of adult femurs of 1.3~1.7 g·cm^−3^ 44,45,46. At this optimal point, the flakes hardness is comparable to that of the human skeleton while the density is far larger. Thus, the ZrO_2_ flakes may be used as biological materials for hip replacements, rosthetic dentistry and dental implantology. This is being pursued in ongoing studies.

## Discussion

ZrO_2_ NPs were synthesized by a simple vapor-phase hydrolysis process^47^. Vickers hardness investigation indicated that both the sintering temperature and compression force played an important role in the determination of the hardness of the ZrO_2_ flakes. AFM studies showed that the surface roughness of the ZrO_2_ flakes gradually decreased with increasing sintering temperature. In addition, the density of the ZrO_2_ flakes was also determined within the context of sintering temperature and compression force. With a systematic variation of these two parameters, hardness and density of the ZrO_2_ nanoflakes were maximized and roughness was minimized simultaneously^48^,^49^. When ZrO_2_ NPs synthesized at 500 °C were pressed into flakes under the compression force of 6 MPa and sintering at 1400 °C, the resulting flakes exhibited an optimal combination of hardness (534.58 gf·mm^−2^), roughness (0.07 μm) and density (4.41 g·cm^−3^).

In conclusion, the experimental results show that both the sintering temperature and compression force played an important role in determining the physical behaviors (hardness, roughness, and density) of ZrO_2_ flakes. Thus, by changing compression force and sintering temperature, the hardness parameters of the ZrO_2_ flakes can be adjusted and comparable to those of human bones, along with a higher density. More importantly, the *in vitro* cytotoxicity and hemolysis evaluation shows that the ZrO_2_ NPs have good biocompatibility. Therefore, it is believed the ZrO_2_ NPs have promising application for bone tissue engineering and regenerative medicine.

## Methods

### Material Preparation

ZrO_2_ NPs were synthesized by a vapor-phase hydrolysis procedure and the experimental apparatus had been reported in a previous study^47^. In brief, the reactor was made of two glass tubes that were externally heated in a vertical furnace. ZrCl_4_ was sublimated at 350 °C and carried by a N_2_ gas (99.9%) into the reaction chamber via the nozzle. Water vapor was introduced into the reaction chamber from around the nozzle by dry air. These two gas streams were mixed rapidly, reacted, and formed ZrO_2_ NPs at atmospheric pressure. Then, the aerosol was cooled by a water jacket tube and filtered at the exit of the reactor for analysis, followed by washing. Three samples of ZrO_2_ NPs were synthesized at different temperatures (400, 500 and 600 °C) and pressed into flakes at different compression force (2, 3, 6, 10, 14 and 18 MPa), which were then subjected to sintering for 6 h at 800, 1000, 1200 and 1400 °C, respectively.

### Cell Culture and Cytotoxicity Evaluation

Human umbilical vein endothelial cells lines (HUVEC) were used to evaluate the cytotoxicity of ZrO_2_ NPs. The cells were cultured in a complete medium Dulbecco’s modified Eagle’s medium (DMEM) containing 10% fetal bovineserum (FBS, GIBCO) and streptomycin/penicillin (100 μg·mL^−1^ Hyclone) at 37 °C in amoisturized 5% CO_2_ incubator. The viability of the treated cells was measured by the Cell Counting Kit-8 (CCK-8) assay. Firstly, HUVEC cells were seeded into a 96-well cell culture plates at the densities of 2 × 10^4^ cells/well, in 100 μL of a complete culture medium at 37 °C for 24 h. Afterward, the ZrO_2_ NPs dispersions were diluted with a fresh medium to the desired concentrations and then added to each well to replace the original culture medium. After another 24 h, the culture medium was removed and replaced by 10 μL of CCK-8 in serum-free media. After incubation for 2 h at 37 °C, the optical density of each well was read at 450 nm on a microplate reader (Spectra Max M2MDC, USA)^37^,^50^,^51^,^52^,^53^,^54^.

Hemolysis Assay of ZrO_2_ NPs were carried out as follows. Kunming mice (5 weeks old) were purchased from Vital River (Beijing, China) and the whole blood was obtained from the mice. All the animals experiments were performed in accordance with the guideline and regulation for the care and use of laboratory animals of Ministry of Science and Technology of People’s Republic of China’s requirements. The Animal Study Committee of the Ministry of Science and Technology of the People’s Republic of China has approved the experiments. Briefly, mice blood were centrifuged and diluted 10 times with PBS to obtain red blood cells (RBCs). Then, 0.2 mL of diluted RBCs were added to 0.8 mL of PBS containing ZrO_2_ NPs at various concentrations (15, 30, 60, 120, 250, 500, and 800 μg·mL^−1^), 0.8 mL of distilled water (positive control), and 0.8 ML of PBS (negative control), respectively. After that, the mixtures were kept at room temperature for 4 h, before they were centrifuged at 12000 rpm for 3 min. Absorbance of the supernatants was measured by a UV-vis spectrophotometer and hemolysis percentage of RBCs was calculated based on the absorbance at 541 nm using equation (1):


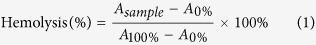


where A_sample_, A_0%_ and A_100%_ are the absorbance of the supernatant of the samples, the negative control, and positive control, respectively.

### Materials Characterizations

The composition, morphologies of the nanoparticles prepared above were characterized by X-ray powder diffraction (XRD, Bruker D8, Cu Kα radiation, λ = 1.54 Å). The size distribution and morphology of the nanoparticles were examined by scanning electron microscopy (SEM, HITACHI, S4800, 15 kV) studies. The hardness of the flakes was evaluated by using a Vickers hardness instrument (UHL Microhardness Testers VMHT. VMH-001) equipped with a cube-corner diamond tip, where the sintered flakes were cleaned by a stream of high-purity nitrogen. To minimize substrate contributions, the indentation experiments were performed in load control, with the load of 50 gf. The loading and unloading speed was 5 × 10^−6^ m·s^−1^, and the time under the peak load was 15 s^48^,^49^,^55^,^56^,^57^,^58^. Ten random tests were performed for each sample to evaluate the material hardness. The surface morphology was analyzed using AFM and the rms roughness was obtained for the ZrO_2_ flakes. The rms roughness data were obtained over an area of 4 μm × 4 μm at five different points of the sample and averaged^57^,^59^,^60^. The density of the flakes was characterized with a precision electronic hydrometer (DH-120M) by equation (2),


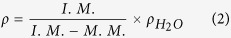


where *ρ* is the density of the flakes, *I.M.* is inherent mass, *M.M.* is measured mass of the flasks and *ρ*_H2O_ is the density of water at 20 °C.

## Additional Information

**How to cite this article**: Wang, J. *et al*. Good Biocompatibility and Sintering Properties of Zirconia Nanoparticles Synthesized via Vapor-phase Hydrolysis. *Sci. Rep.* **6**, 35020; doi: 10.1038/srep35020 (2016).

## Supplementary Material

Supplementary Information

## Figures and Tables

**Table 1:** Physical hardness (gf·mm^−2^) of ZrO_2_ flakes.

C. Force	S. Temp	2 MPa	3 MPa	6 MPa	10 MPa	14 MPa	18 MPa
Hardness							
Syn. Temp							
400 °C	800 °C	150.28	411.64	200.55	286.30	332.89	324.56
	1000 °C	188.00	460.30	311.34	299.65	431.36	420.47
	1200 °C	543.85	603.25	440.13	467.03	460.85	454.70
	1400 °C	450.51	477.59	462.29	452.65	419.63	405.69
500 °C	800 °C	99.71	284.30	233.39	248.84	266.57	262.87
	1000 °C	103.38	331.60	387.94	375.85	357.65	356.67
	1200 °C	548.40	562.00	476.36	473.04	468.07	458.26
	1400 °C	383.63	542.62	**534.58**	477.21	468.65	460.44
600 °C	800 °C	195.33	343.27	133.21	263.46	251.32	246.58
	1000 °C	104.90	252.67	232.78	371.88	446.96	425.60
	1200 °C	575.50	621.30	492.09	502.75	530.99	408.04
	1400 °C	503.22	765.98	488.67	463.27	417.01	412.79

The ZrO_2_ flakes prepared by compression of ZrO_2_ NPs synthesized at 400, 500 and 600 °C under 2, 3, 6, 10, 14 and 18 MPa and sintering at 800, 1000, 1200 and 1400 °C (C. Force: compression forces, S. Temp: sintering temperature, Syn. Temp: Synthesis temperature).

**Table 2:** Surface roughness (μm) of ZrO_2_ flakes.

C. Force	S. Temp	2 MPa	3 MPa	6 MPa	10 MPa	14 MPa	18 MPa
Rms. Roughness							
Syn. Temp							
400 °C	800 °C	0.22	0.40	0.45	0.29	0.21	0.22
	1000 °C	0.18	0.32	0.23	0.23	0.16	0.21
	1200 °C	0.17	0.29	0.27	0.22	0.22	0.19
	1400 °C	0.14	0.13	0.21	0.10	0.07	0.12
500 °C	800 °C	0.30	0.47	0.37	0.56	0.65	0.22
	1000 °C	0.19	0.25	0.30	0.33	0.26	0.21
	1200 °C	0.18	0.33	0.26	0.34	0.24	0.15
	1400 °C	0.05	0.16	**0.07**	0.07	0.07	0.08
600 °C	800 °C	0.15	0.51	0.28	0.47	0.29	0.20
	1000 °C	0.25	0.13	0.25	0.26	0.23	0.19
	1200 °C	0.22	0.24	0.38	0.27	0.21	0.11
	1400 °C	0.07	0.09	0.08	0.13	0.13	0.06

The ZrO_2_ flakes prepared by compression of ZrO_2_ NPs synthesized at 400, 500 and 600 °C under 2, 3, 6, 10, 14 and 18 MPa and sintering at 800, 1000, 1200 and 1400 °C.

**Table 3:** Density (g·cm^−3^) of ZrO_2_ flakes.

C. Force	Syn. Temp	2 MPa	3 MPa	6 MPa	10 MPa	14 MPa	18 MPa
Density							
S. Temp							
800 °C	400 °C	3.47	3.80	4.57	3.68	3.78	4.95
	500 °C	4.38	3.09	3.20	4.11	5.69	5.86
	600 °C	3.50	3.85	2.59	3.87	6.05	5.60
1000 °C	400 °C	3.58	3.56	4.55	4.45	5.58	5.28
	500 °C	4.04	4.63	4.24	5.66	5.55	6.33
	600 °C	3.76	3.57	5.44	5.94	5.29	6.38
1200 °C	400 °C	5.53	4.43	4.42	3.64	5.52	4.56
	500 °C	4.85	5.43	7.40	4.87	6.15	5.28
	600 °C	3.64	3.40	3.86	5.40	4.68	2.58
1400 °C	400 °C	3.94	3.90	3.18	5.36	5.62	5.28
	500 °C	2.91	3.79	**4.41**	4.60	5.15	4.87
	600 °C	3.09	2.24	5.23	4.77	3.67	5.12

The ZrO_2_ flakes prepared by compression of ZrO_2_ NPs synthesized at 400, 500 and 600 °C under 2, 3, 6, 10, 14 and 18 MPa and sintering at 800, 1000, 1200 and 1400 °C.

## References

[b1] Holzapfel B. M. . How smart do biomaterials need to be? A translational science and clinical poin t of view. Adv. Drug Delivery Rev. 65, 581–603 (2013). 10.1016/j.addr.2012.07.009 22820527

[b2] He G. P. . Addition of Zn to the ternary Mg–Ca–Sr alloys significantly improves their antibacterial properties. J. Mater. Chem. B 3, 6676–6686 (2015). 10.1039/C5TB01319D PMC4675164 26693010

[b3] Xu J., Liu L. L., Munroe P. & Xie Z. H. Promoting bone-like apatite formation on titanium alloys through nanocrystalline tantalum nitride coatings. J. Mater. Chem. B 3, 4082–4094 (2015). 10.1039/c5tb00236b 32262630

[b4] Roy M., Balla V. K., Bandyopadhyay A. & Bose S. MgO-Doped Tantalum Coating on Ti: Microstructural Study and Biocompatibility Evaluation. Mater. Interfaces 4, 577–580 (2012). 10.1021/am201365e PMC3288316 22248182

[b5] Kelly J. R. & Denry I. Stabilized zirconia as a structural ceramic: An overview. Dent. Mater. 24, 289–298 (2008). 17624420 10.1016/j.dental.2007.05.005

[b6] Gu X. Y., Li X., Liu Q., Chen Y. X. & Li Y. X. Effects of Calcium Acetate on the Hydrothermal Synthesis of Tetragonal-Phase ZrO_2_ (3Y) Nanocrystals. Rare Met. Mater. Eng. 38, 1044–1066 (2009).

[b7] Liu X. Y., Chu P. K. & Ding C. X. Surface nano-functionalization of biomaterials. Mater. Sci. Eng. R 70, 275–302 (2010).

[b8] Lee H. S., Huy C. N., Pham T. T. & Shin E. W. ZrO_2_-impregnated red mud as a novel catalyst for steam catalytic cracking of vacuum residue. Fuel 165, 462–467 (2016).

[b9] Harju H., Lehtonen J. & Lefferts L. Steam reforming of *n*-butanol over Rh/ZrO_2_ catalyst: role of 1-butene and butyraldehyde. Appl. Catal. B 182, 33–46 (2016).

[b10] Goscianska J., Ziolek M., Gibson E. & Daturi M. Meso–macroporous zirconia modified with niobia as support for platinum—Acidic and basic properties. Catal. Today 152, 33–41 (2010).

[b11] Ward D. A. & Ko E. I. Preparing Catalytic Materials by the Sol-Gel Method. Ind. Eng. Chem. Res. 34, 421–433 (1995).

[b12] Li Y. W. . Effect of calcium salts on isosynthesis over ZrO_2_ catalysts. J. Mol. Catal. A: Chem. 175, 267–275 (2001).

[b13] Viazzi C., Bonino J. P. & Ansart F. Synthesis by sol-gel route and characterization of Yttria Stabilized Zirconia coatings for thermal barrier applications. Surf. Coat. Technol. 201, 3889–3893 (2006).

[b14] Mazzoni A. D. & Conconi M. S. Study of carbonitriding reactions of zirconia. Synthesis of Zr (C, N, O) phases and β-type zirconium oxynitrides. Ceram. Int. 30, 23–29 (2004).

[b15] Ravichandran K. S., An K., Dutton R. E. & Semiatin S. L. Thermal conductivity of plasma-sprayed monolithic and multilayer coatings of alumina and yttria-stabilized zirconia. J. Am. Ceram. Soc. 82, 673–682 (1999).

[b16] Korotcenkov G., Han S. D. & Stetter J. R. Review of Electrochemical Hydrogen Sensors. Chem. Rev. 109, 1402–1433 (2009). 19222198 10.1021/cr800339k

[b17] Subbarao E. C. & Maiti H. S. Oxygen sensors and pumps. Adv. Ceram. 24, 731–747 (1988).

[b18] Manicone P. F., Iommetti P. R. & Raffaelli L. An overview of zirconia ceramics: Basic properties and clinical applications. J. Dent. 35, 819–826 (2007). 17825465 10.1016/j.jdent.2007.07.008

[b19] Wang Y. Q. & Sayre G. Commercial thermal barrier coatings with a double-layer bond coat on turbine vanes and the process repeatability. Surf. Coat. Technol. 203, 2186–2192 (2009).

[b20] Thamaraiselvi T. V. & Rajeswari S. Biological evaluation of bioceramic materials. Trends Biomater. Artif. Organs 18, 9–17 (2004).

[b21] He G. P. . Addition of Zn to the ternary Mg–Ca–Sr alloys significantly improves their antibacterial properties. J. Mater. Chem. B 3, 6676–6689 (2015). 10.1039/C5TB01319D PMC4675164 26693010

[b22] Xu J., Liu L. L., Munroe P. & Xie Z. H. Promoting bone-like apatite formation on titanium alloys through nanocrystalline tantalum nitride coatings. J. Mater. Chem. B 3, 4082–4094 (2015). 10.1039/c5tb00236b 32262630

[b23] Xia B., Duan L. Y. & Xie Y. C. ZrO_2_ nanopowders prepared by low-temperature vapor-phase hydrolysis. J. Am. Ceram. Soc. 83, 1077–1080 (2000).

[b24] Fang Z. T. & Dixon D. A. Hydrolysis of ZrCl_4_ and HfCl_4_: The Initial Steps in the High-Temperature Oxidation of Metal Chlorides to Produce ZrO_2_ and HfO_2_. J. Phys. Chem. C 117, 7459–7474 (2013).

[b25] Suyama Y., Mizobe T. & Kato A. ZrO_2_ Powders Produced by Vapor Phase Reaction. Ceramurgia Int. 3, 141–146 (1977).

[b26] Mazdiyasni K. S., Lynch C. T. & Smith J. S. Preparation of Ultra-High-Purity Submicron Refractory Oxides. J. Am. Ceram. Soc. 48, 372–375 (1965).

[b27] Tok A. I. Y., Boey F. Y. C., Duand S. W. & Wong B. K. Flame spray synthesis of ZrO_2_ nano-particles using liquid precursors. Mater. Sci. Eng. B 130, 114–119 (2006).

[b28] Esparza-Ponce H. E., Reyes-Rojas A., Antúnez-Flores W. & Miki-Yoshida M. Synthesis and characterization of spherical calcia stabilized zirconia nano-powders obtained by spray pyrolysis. Mater. Sci. Eng. A 343, 82–88 (2003).

[b29] Burleson D. J., Roberts J. T., Gladfelter W. L., Campbell S. A. & Smith R. C. A Study of CVD Growth Kinetics and Film Microstructure of Zirconium Dioxide from Zirconium Tetra-*tert*-Butoxide. Chem. Mater. 14, 1269–1276 (2002).

[b30] Chandra N. . Synthesis and characterization of nano-sized zirconia powder synthesized by single emulsion-assisted direct precipitation. J. Colloid Interface Sci. 342, 327–332 (2010). 19942226 10.1016/j.jcis.2009.10.065

[b31] Wang S. Y., Li X. A., Zhai Y. C. & Wang K. M. Preparation of homodispersed nano zirconia. Powder Technol. 168, 53–58 (2006).

[b32] Rezaei M., Alavi S. M., Sahebdelfar S. & Yan Z. F. Tetragonal nanocrystalline zirconia powder with high surface area and mesoporous structure. Powder Technol. 168, 59–63 (2006).

[b33] Hsu Y. W., Yang K. H., Chang K. M., Yeh S. W. & Wang M. C. Synthesis and crystallization behavior of 3 mol% yttria stabilized tetragonal zirconia polycrystals (3Y-TZP) nanosized powders prepared using a simple co-precipitation process. J. Alloys Compd. 509, 6864–6870 (2011).

[b34] Tahmasebpour M., Babaluo A. A. & Aghjeh M. K. R. Synthesis of zirconia nanopowders from various zirconium salts via polyacrylamide gel method. J. Eur. Ceram. Soc. 28, 773–778 (2008).

[b35] Heshmatpour F. & Aghakhanpour R. B. Synthesis and characterization of superfine pure tetragonal nanocrystalline sulfated zirconia powder by a non-alkoxide sol–gel route. Adv. Powder Technol. 23, 80–87 (2012).

[b36] Dwivedi R., Maurya A., Verma A., Prasada R. & Bartwal K. S. Microwave assisted sol–gel synthesis of tetragonal zirconia nanoparticles. J. Alloys Compd. 509, 6848–6851 (2011).

[b37] Yin W. Y., Yan L., Yu J. & Tian G. High-Throughput Synthesis of Single-Layer MoS_2_ Nanosheets as a Near-Infrared Photothermal-Triggered Drug Delivery for Effective Cancer Therapy. ACS nano. 8, 6922–6933 (2014). 24905027 10.1021/nn501647j

[b38] Song M. M., Song W. J., Bi H. & Wang J. Cytotoxicity and cellular uptake of iron nanowires. Biomaterials 31, 1509–1517 (2010). 19945156 10.1016/j.biomaterials.2009.11.034

[b39] Cho S. M. . Multifunctional Composite Coating as a Wear-Resistant Layer for the Bearing in Total Hip Joint Replacement. ACS Appl. Mater. Interfaces 5, 395–403 (2013). 23249257 10.1021/am302452f

[b40] Clausell C., Barba A., Nuno L. & Jarque J. C. Effect of average grain size and sintered relative density on the imaginary part – *μ*″ of the complex magnetic permeability of (Cu_0.12_Ni_0.23_Zn_0.65_) Fe_2_O_4_ system. Ceramics International 42, 4256–4261 (2016).

[b41] Arias D. F., Arango Y. C. & Devia A. Study of TiN and ZrN thin films grown by cathodic arc technique. Applied Surface Science 253, 1683–1690 (2006).

[b42] Liu B., Xiao H. Y., Zhang Y. W., Aidhy D. S. & Weber W. J. Investigation of oxygen point defects in cubic ZrO_2_ by density functional theory. Computational Materials Science 92, 22–27 (2014).

[b43] Negara M. A., Goel N., Bauza D., Ghibaudo G. & Hurley P. K. Interface state densities, low frequency noise and electron mobility in surface channel In_0.53_Ga_0.47_ As *n*-MOSFETs with a ZrO_2_ gate dielectric. Microelectronic Engineering 88, 1095–1097 (2011).

[b44] Wen J. G., Liu Y., Liao X., Shi L. J. & Liu J. Y. An investigation of bone density among children aged over 5 years old in Shenyang city. Chinese Journal of Woman and Child Health Research 19, 15–16 (2008).

[b45] Zhang J. H., Wang L. & Wang Y. L. Investigation of bone mineral density in punching and welding workers. Chin J Osteoporos 21, 706–707 (2015).

[b46] Zhang M. M., Li Y. G. & Liu Y. Study on the influencing factors for bonemineral density among 24831 people in Changchun. Chin J Osteoporos 16, 125–127 (2010).

[b47] Wang Q. & Li C. H. Controllable synthesis of zirconia nano-powders using vapor-phase hydrolysis and theoretical analysis. J. Mater. Chem. A 2, 1346–1352 (2014).

[b48] Julietta V. R. . Physicochemical Investigation of Pulsed Laser Deposited Carbonated Hydroxyapatite Films on Titanium. ACS Appl. Mater. Interfaces 1, 1813–1820 (2009). 20355798 10.1021/am900356e

[b49] Oliver W. C. & Pharr G. M. An improved technique for determining hardness and elastic modulus using load and displacement sensing indentation experiments. J. Mater. Res. 7, 1564–1583 (1992).

[b50] Zhang F. . A Novel High Mechanical Property PLGA Composite Matrix Loaded with Nanodiamond–Phospholipid Compound for Bone Tissue Engineering. ACS Appl. Mater. Interfaces 8, 1087–1097 (2016). 26646188 10.1021/acsami.5b09394

[b51] Chandra V. S., Baskar G., Suganthi R. V. & Elayaraja K. Blood Compatibility of Iron-Doped Nanosize Hydroxyapatite and Its Drug Release. ACS Appl. Mater. Interfaces 4, 1200–1210 (2012). 22316071 10.1021/am300140q

[b52] Zhang Z. Q. . The influence of UV irradiation on the biological properties of MAO-formed ZrO_2_. Colloids. Surf. B 89, 40–47 (2012). 10.1016/j.colsurfb.2011.08.020 21920713

[b53] Catauro M. . Biological response of human mesenchymal stromal cells to titanium grade 4 implants coated with PCL/ZrO_2_ hybrid materials synthesized by sol–gel route: *in vitro* evaluation. Mater. Sci. Eng. C 45, 395–401 (2014). 10.1016/j.msec.2014.09.007 25491844

[b54] Wang Y., Yu H. J., Chen C. Z. & Zhao Z. H. Review of the biocompatibility of micro-arc oxidation coated titanium alloys. Materials and Design. 85, 640–652 (2015).

[b55] Garvie R. C. & Nicholson P. S. Phase Analysis in Zirconia Systems. J. Am. Ceram. Soc. 55, 303–305 (1972).

[b56] Cabibbo M. . An international round-robin calibration protocol for nanoindentation measurements. Micron. 43, 215–222 (2012). 21890366 10.1016/j.micron.2011.07.016

[b57] Dorcioman G., Socol G., Craciun D. & Argibay N. Wear tests of ZrC and ZrN thin films grown by pulsed laser deposition. Appl. Surf. Sci. 306, 33–36 (2014).

[b58] Craciun D. . Thin and hard ZrC/TiN multilayers grown by pulsed laser deposition. Surf. Coat. Technol. 205, 5493–5496 (2011).

[b59] Craciun D. . Characteristics of ZrC/ZrN and ZrC/TiN multilayers grown by pulsed laser deposition. Appl. Surf. Sci. 257, 5332–5336 (2011).

[b60] Thornton A. J. High rate thick film growth. Ann. Rev. Mater. Sci. 7, 239–260 (1977).

