# Supplementary material for: Good Biocompatibility and Sintering Properties of Zirconia Nanoparticles Synthesized via Vapor-phase Hydrolysis
Source: Sci Rep. 2016 Oct 11;6:35020. doi: 10.1038/srep35020 (PMC5057113; doi:10.1038/srep35020)
Supplement: Supplementary Information [file srep35020-s1.doc]

**Supporting information**

**SUBJECT AREAS: PHYSICAL SCIENCES, CHEMISTRY**

**Good Biocompatibility and Sintering Properties of Zirconia Nanoparticles Synthesized via Vapor-phase Hydrolysis**

JigangWang^1,2,§^,WenyanYin^3,§^,Xiao He^3^,Qiang Wang^1,2,*^,Ming Guo^1^﹠Shaowei Chen^4,*^

^1^Laboratory for Micro-sized Functional Materials &College of Elementary Education, Capital Normal University, Beijing, 100048, PR China.

^2^Department of Chemistry, Capital Normal University, Beijing, 100048, PR China.

^3^Key Laboratory for Biomedical Effects of Nanomaterials and Nanosafety Institute of High Energy Physics, Chinese Academy of Sciences, Beijing, 100049, China.

^4^Department of Chemistry and Biochemistry, University of California, Santa Cruz, CA 95064, USA.

^*^Correspondence and requests for materials should be addressed to Q.W. ([qwchem@gmail.com](mailto:qwchem@gmail.com))

^§^The authors contributed equally to this work.


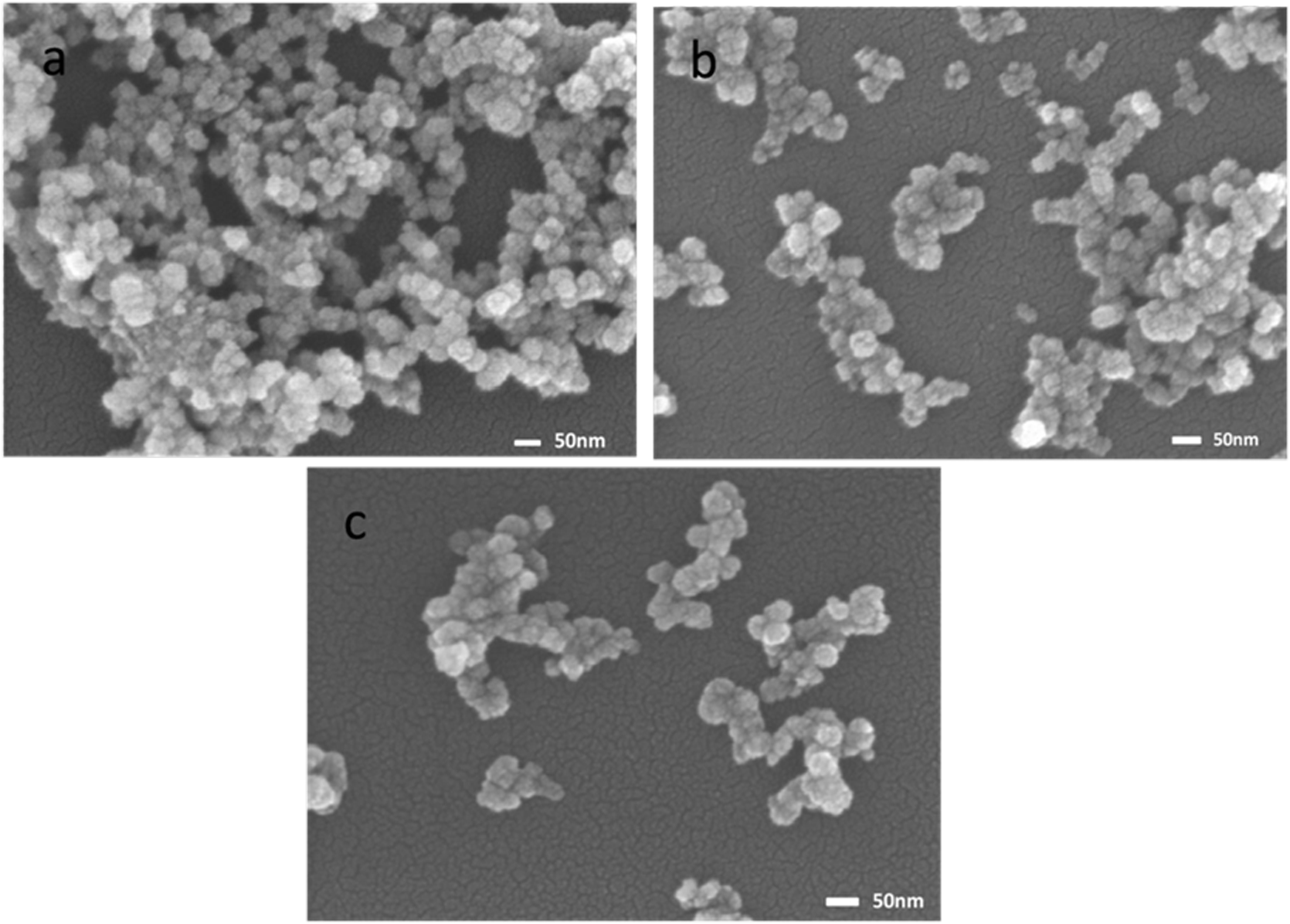


**Supporting Figure S1.** SEM images of ZrO_2_ NPs. ZrO_2_ NPs synthesized at different temperatures (a) 400 °C, (b) 500 °C, and (c) 600 °C.


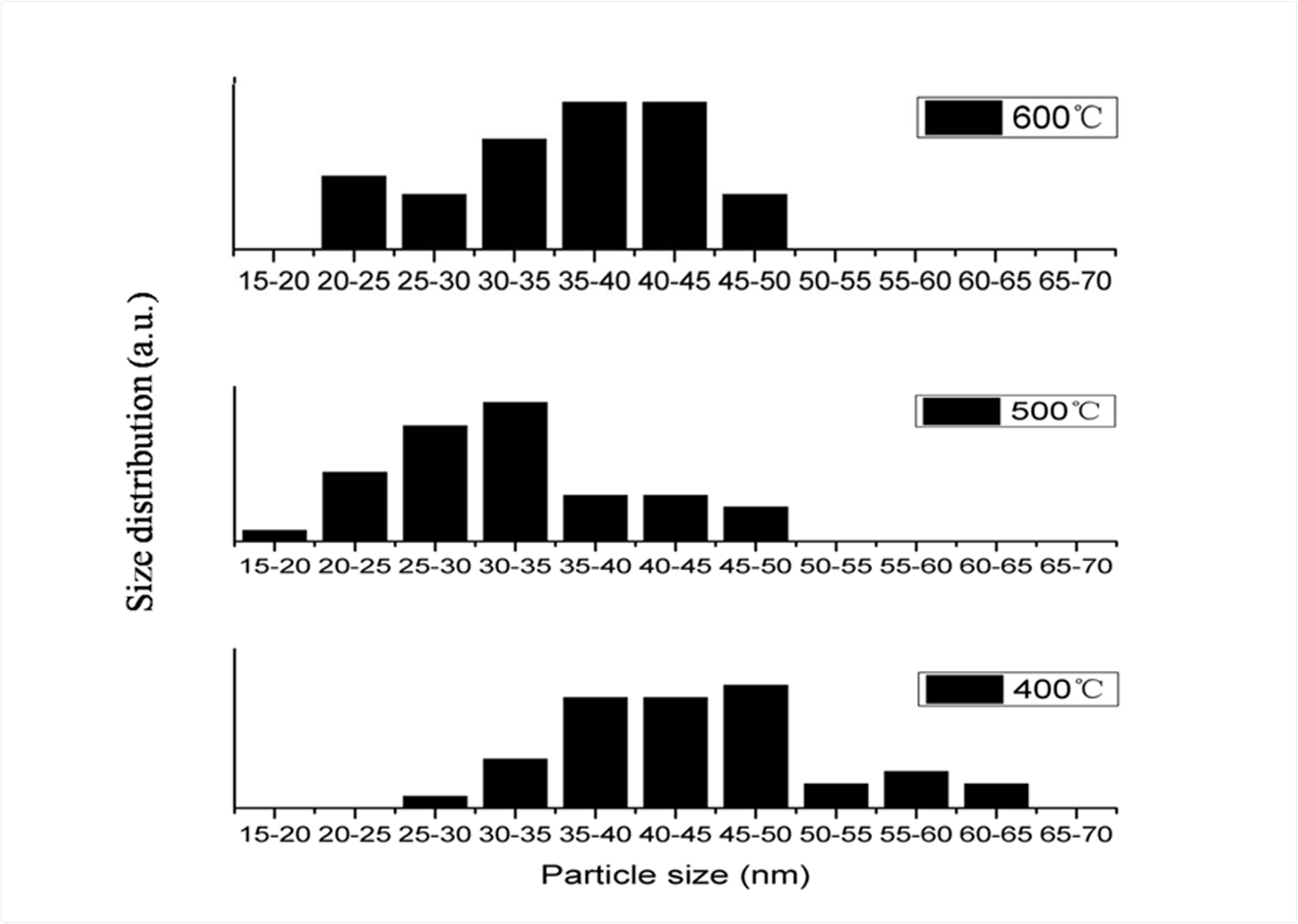


**Supporting Figure S2.** Particle size distribution of ZrO_2_ NPs. ZrO_2_ NPs synthesized with different temperatures.


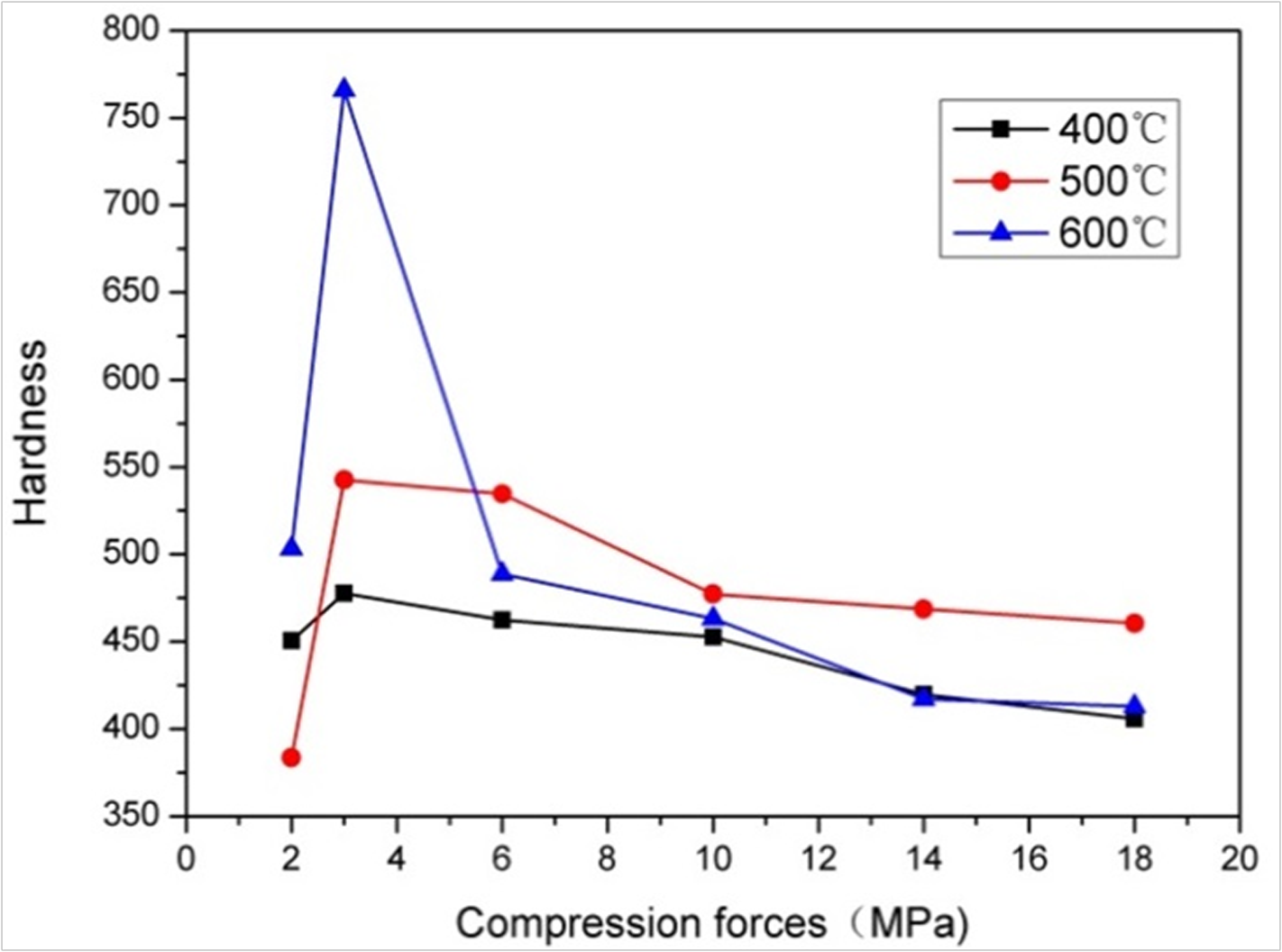


**Supporting Figure S3.** Hardness of ZrO_2_ flakes. The ZrO_2_ flakes were prepared by sintering at 1400 °C under different compression forces.


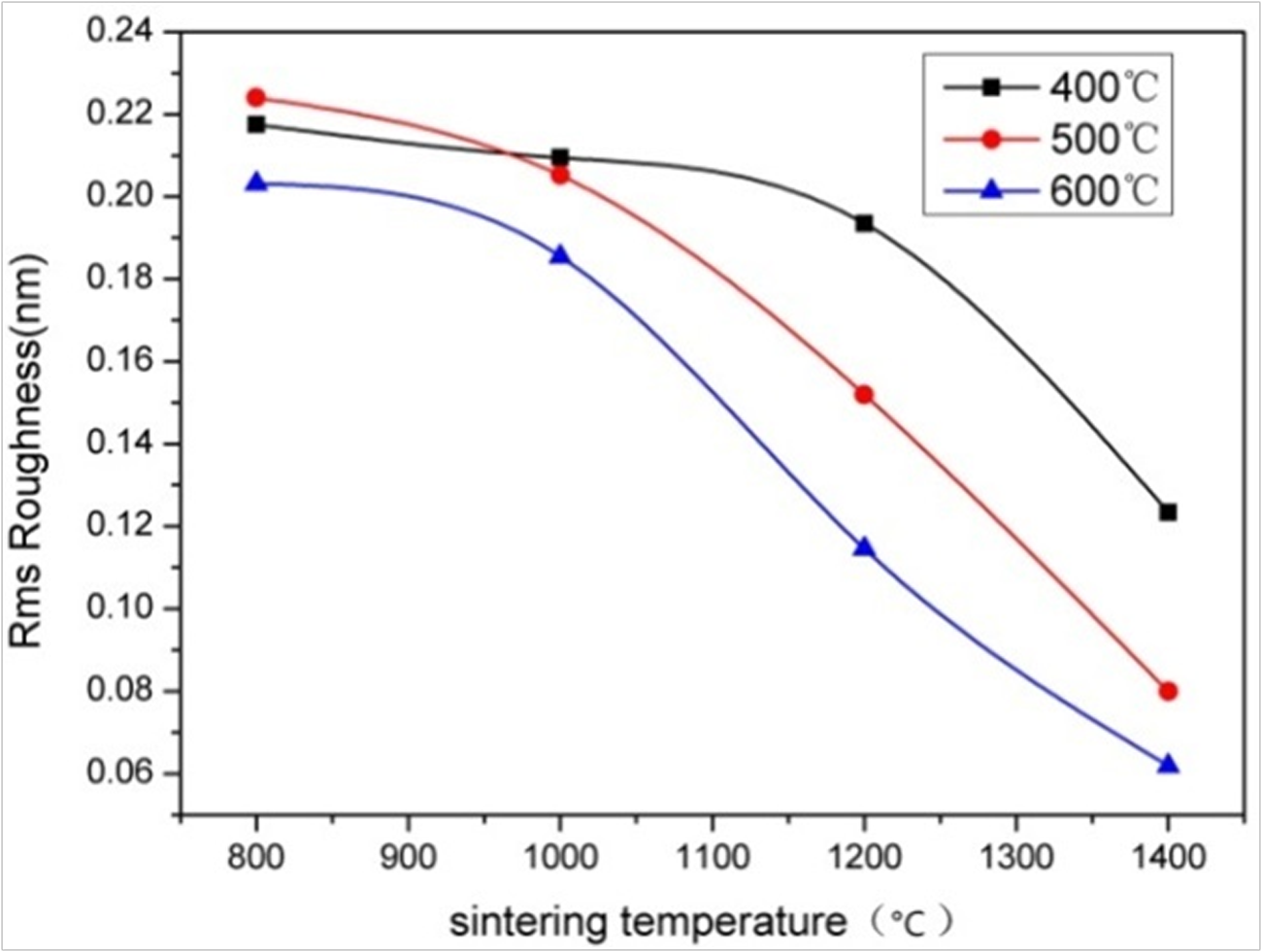


**Supporting Figure S4.** Surface roughness of ZrO_2_ flakes. The ZrO_2_ flakes pressed at 18 MPa, under different sintering temperatures.
